# Evaluating the Involving Relationships between Temperament and Motor Coordination in Early Childhood: A Prognostic Measurement

**DOI:** 10.3390/brainsci11030333

**Published:** 2021-03-06

**Authors:** Maria Sofologi, Sophia Koulouri, Despina Moraitou, Georgia Papantoniou

**Affiliations:** 1 Psychology Laboratory, Department of Early Childhood Education, School of Education, University of Ioannina, 45100 Ioannina, Greece; sophiakoulouri@gmail.com (S.K.); gpapanto@uoi.gr (G.P.); 2 Institute of Humanities and Social Sciences, University Research Centre of Ioannina (U.R.C.I.), 45100 Ioannina, Greece; 3 Laboratory of Psychology, Section of Experimental and Cognitive Psychology, School of Psychology, Aristotle University of Thessaloniki, 54124 Thessaloniki, Greece; demorait@psy.auth.gr; 4 Laboratory of Neurodegenerative Diseases, Center for Interdisciplinary Research and Innovation (CIRI—AUTH) Balkan Center, Buildings A & B, Aristotle University of Thessaloniki, 57001 Thessaloniki, Greece

**Keywords:** early childhood, effortful control, motor coordination, negative affectivity, surgency/extraversion

## Abstract

The main aim of the present study was the evaluation of dynamic relationships between dimensions of temperament and motor coordination in 3–7-year-old children in Greece. More specifically, the main objectives of the current study were the test outcomes regarding the psychometric properties (structural validity and internal consistency) of the Greek versions of (a) the Child Behavior Questionnaire—very short format (CBQ—VSF), and (b) the Developmental Coordination Disorder Questionnaire (DCDQ). For the purposes of the present study, 231 parents (202 women and 29 men), aged 23–53 years (mean (M) = 36.7 and standard deviation (SD) = 5.4) completed the aforementioned questionnaires. The sample consisted of 231 children (110 girls and 121 boys) aged 3–7 years (M = 4.75 years and SD = 1.30). For the DCDQ, the confirmatory factor analysis revealed three factors consistent with the factors that emerged from the constructs, with strong internal consistency reliability. Furthermore, regarding the CBQ—VSF, which measures the dimensions of temperament, the implementation of the confirmatory factor analysis indicated three factors and satisfactory internal consistency reliability, as well. Finally, path analysis revealed that temperamental effortful control, which mirrors both inhibitory and self-regulatory capacity, has a positive effect on motor coordination.

## 1. Introduction

Temperament research on children is derived from the fields of clinical and developmental psychology, psychiatry, pediatrics, and education. Temperament refers to individual characteristics or traits having a biological or genetic basis, which determine an individual’s emotional and motor reactivity and plays a significant role both in subsequent social interactions and social functioning [1]. Specifically, the construct of temperament is commonly viewed as the basic organization of personality, which is observable as early as infancy and becomes elaborated over the course of development as the individual’s skills, abilities, cognitions, and motivations become more sophisticated [2,3]. It is essential to mention that as a result of a developmental continuum, these dimensions are morphed over time through maturity and experience, enhanced by scenarios or cognitive schemas from everyday life and everyday activities [4]. In particular, temperament functions as a dynamic mechanism that interprets how individuals contribute to their development in a particular environment. Additionally, through a two-way interaction between inherent and temperamental traits and external experiences and circumstances, a harmonic balance among individuals and their environment is produced [5].

It is an axiom that the core dimensions of temperament and optimal method for assessment remain as sources to emphasize on a multi-facet personality research discussion. Nevertheless, the moderate stability of most temperamental traits and the strong influence of the amalgamation between genetic and environmental factors have been well established, along with temperament’s association with childhood psychiatric disorders. Additionally, a major stimulus for temperament research has emerged from its obvious clinical relevance and clinical observations [6]. As a result, in the last four decades, a literature review reveals different fundamental theoretical models supporting the relationship of temperament characteristics and a variety of typologies for future behaviors. The research community is looking for behavioral characteristics marking basic environmental, psychophysiological, and genetic processes. There is a research consensus regarding some key significant components of temperamental traits, including their manifestation early in life, genetic influence, and longitudinal stability over the lifespan. The evaluation process of temperament can be divided into different temperament research traditions: (a) psychological theoretical approaches, (b) genetic inheritance as essential for all temperament traits, and (c) neurodevelopmental approaches.

According to the first tradition, psychological development is not only influenced by the child’s temperament and its facets, but also by the parental coping strategies to specific temperament manifestations, to an equally significant extent [7]. According to Thomas and Chess (1987), changes in the social environment could cause changes in temperament [8]. Furthermore, they support the hypothesis that the difficult infant is at risk for behavior problems. Regarding the second theoretical tradition, human existence and experience are always simultaneously both biological and social, and an adequate explanation must involve both. More specifically, according to Kagan (1994) [9], the behavioral reactions to unfamiliar events are basic phenomena in all vertebrates. Four-month-old infants who show a low threshold to become distressed and motorically aroused to unfamiliar stimuli are more likely to become fearful and subdued during early childhood than others, whereas infants who show a high arousal threshold are more likely to become bold and sociable. Finally, according to the neurodevelopmental tradition, temperament is characterized by differences in reactivity and self-regulation, which are influenced over time by heredity, maturity, and life experiences [10]. The concept of reactivity refers to biological stimulation, motor activation, emotionality autonomy, and endocrine systems. Self-regulation refers to the processes that regulate reactivity, such as attention, inhibition, and self-reassurance.

### 1.1. Developing Perspectives of Temperament: The Three General Temperamental Dimensions

In an attempt to illustrate empirical and conceptual considerations, temperament is an open dynamic system being influenced by environment interactions [11]. Rothbart further suggested that the dynamic role of caretakers will be particularly important for the development of temperamentally vulnerable children [12]. Finally, she strongly supported the developmental nature of temperament, according to which the developmental change could affect reactive and regulatory processes in a number of ways. Furthermore, in the research with infants aged 3 up to 12 months, the following three broad factors concerning temperament were revealed: (1) Surgency/Extraversion, (2) Negative Affectivity, and (3) Regulatory Capacity/Effortful Control, with additional factors emerging in the oldest populations [13]. The first two temperamental dimensions are concerned with emotional reactivity, while the third is related to individual differences regarding self-regulation and control of reactivity [14]. Specifically, the first factor Surgency/Extraversion includes positive anticipation, impulsivity, and increased levels of activity. Furthermore, it is of vital importance to underline the fact that it reflects the degree to which a child is generally happy, active, and enjoys vocalizing and seeking stimulation. Increased levels of smiling and laughter are observed in babies with high levels in Surgency/Extraversion, whereas 10- to 11-year-olds with higher levels of Surgency/Extraversion are less likely to develop internalizing problems such as shyness and low self-esteem [15]. The second factor of the temperament theory, Negative Affectivity, includes fear, frustration, sadness, dysthymia, and anger [15]. Additionally, anger and frustration are observed in the early second to third month. Anger and frustration, together, predict both externalizing and internalizing difficulties. Fear, as evidenced by behavioral inhibition, is seen as early as 7–10 months of age and later predicts children’s fearfulness and lower levels of aggression [5]. Finally, Effortful Control includes the focusing and shifting of attention, inhibitory control, perceptual sensitivity, and a low threshold for pleasure [5]. This factor reflects the degree to which a child can focus attention and engage in planning. It is an axiom that Effortful Control mirrors the ability to delay or inhibit a prepotent response, often in favor of a subdominant response, and is considered the regulatory dimension of temperament [16]. Reactive temperament appears early in infancy and stabilizes into childhood. Furthermore, Developing Effortful Control is considered to be caused by the development in the executive attention system of the brain [17]. Effortful Control is high when six- to seven-year-olds tend to be more empathetic and lower in aggressiveness. It can also be considered as the ability to control one’s actions, emotions, and attention [18]. Additionally, in an attempt to shed light on gender differences, the literature review seems to reveal small gender differences in temperament [19]. For example, consistent gender differences favoring girls were found within the factor of effortful control. More specifically, girls display a better ability to control inappropriate responses and behaviors than boys, in association with overall better ability to regulate their impulses or allocate their attention in comparison with boys [19]. Also, several dimensions within surgency showed small to moderate gender differences favoring boys. The literature review reveals a small positive gender difference favoring boys in high-intensity pleasure, which represents the amount of pleasure, derived from high stimulus intensity, rate, complexity, novelty, and incongruity, and might include rough-and-tumble play or being in crowds of people [20]. Finally, regarding the negative affectivity, only negligible gender differences were revealed, showing that boys and girls do not differ in the extent to which they are difficult, emotional, or suitable [21]. Undoubtedly, both an increased tendency to experience negative emotions and a decreased ability to regulate emotional responses appear common to many childhood psychiatric disorders—although, not surprisingly, the links between other temperamental traits and psychopathology vary by disorder. According to the above-mentioned theoretical approaches and taking into consideration the researches that include dimensions such as activity level, attentional control, and impulsivity as temperament dimensions, it should come as no surprise that the association between temperament and Developmental Coordination Disorder (DCD) is strong enough to warrant the speculation that the disorder is perhaps better understood dimensionally.

### 1.2. Characteristics of the Developmental Coordination Disorder (DCD)

Developmental Coordination Disorder (DCD) is one of the most common disorders found in school-age children [19]. It is a medically preferred term, used in an attempt to describe children with developmental coordination difficulties. It is a chronic and usually permanent condition observed in children’s population and is characterized by motor dysfunction that impedes the child’s daily activities and academic success [20,21]. At home, a child with DCD diagnosis may lack the ability to be independent in self-care tasks such as dressing [22]. In physical activities, children with DCD may look awkward when walking and running and be less proficient than their peers in ball skills, agility, and balance-based activities [23]. In the classroom, children with DCD experience particular difficulties with pencil control, handwriting acquisition, organization, and legibility of written work [22]. All these difficulties have raised concerns among educators, psychologists, and therapists about the negative effects of DCD on the emotional and psychological well-being of children [24]. It should be underlined that a significant characteristic of the disorder is that the motor impairment should not be caused by or be presented along with the symptoms of a recognizable neurological problem. That is, the child should not have muscle tone disorders (ataxia or spasticity), loss of consciousness, or involuntary movements [20]. Furthermore, when describing children with developmental coordination disorder, it is of vital importance to recognize its heterogeneity. More specifically, some children may have difficulties in various areas, while others may have problems only with specific activities [25]. On the other hand, researchers underline the fact that motor deficits and coordination impairments commonly continue in adulthood and may be expressed as slowness and avoidance from participating in motor activities and other social activities, as well. As a result, it seems that impairment in motor skills may have significant emotional and social impact [26].

The literature review reveals that there are sporadic surveys concerning the relation between infant temperament and neurobehavioral measures. The research community have consistently identified neurodevelopmental disorders as being linked to specific temperament configurations [26]. Moreover, the literature review reveals sporadic evidence concerning the relation between temperament and neural markers, including poor motor skills and sensory differences [27]. Specifically, researchers suggested a significant connection between motor competence and temperamental attentional processes in 1- month-old infants through principal component analysis [28]. It seems clear that when studying temperament, the research community have focused on the emotion and attention systems and have been less concerned with the role of sensory and motor regulation, which is a regulatory process operating across multiple sensory domains [29]. In this vein, Nakagawa Miyachi, Sukigara, and Seo analyzed data from 1892 infants in order to examine the above-mentioned relationship as part of the Japan Environment, a Children’s Study conducted by the Japanese Ministry of the Environment [30]. Their research findings are closely aligned and consistent with the theoretical idea that surgent tendencies should be viewed as an accelerator toward action in infants, with inhibitory tendencies such as fear (one of the subscales for Νegative Affectivity) and Effortful Control as brakes [14]. Additionally, Nakagawa et al. [30] investigated the relations between temperament and motor coordination in healthy 3-year-old children and revealed that effortful control affects motor coordination, while Surgency affects one of the motor coordination components, namely control during movement [25]. Negative affectivity was found to affect none of the motor coordination components.

## 2. The Present Study

Similarly, under the theoretical aegis of the three temperamental factors of Rothbart’s theory, the primary aim of the present study was the evaluation of the relations between temperamental traits and indicators of the Developmental Coordination Disorder (DCD) in early childhood. The second aim of the current study was the evaluation of the psychometric properties (structural validity and internal consistency reliability) of (a) the Greek version of the Child Behavior Questionnaire—Very Short Form (CBQ—VSF), and (b) the Greek version of the Questionnaire for the Developmental Coordination Disorder Questionnaire (DCDQ). Additionally, a third aim of the present study, based on the literature review of Cosentino-Rocha and Linhares (2013) [31], Else-Quest, Hyde, Goldsmith, and Van Hulle (2006) [32], and Walker, Berthelsen, and Irving (2001) [33], was the evaluation of gender differences in the three components of temperament (extraversion/outburst of emotions, negative mood, and effortful control) [34].

## 3. Subjects and Methods

For the purpose of the present study, 231 parents participated (202 women and 29 men), aged 23–53 years (mean (M) = 36.7 and standard deviation (SD) = 5.4). Demographic characteristics of professional and educational profile of parents are presented in Table 1. Furthermore, for the purpose of the present study, 231 young children (110 girls and 121 boys), aged from 3 to 7 years (M = 4.75 years and SD = 1.30), were rated by one of their parents. The availability of the parent was considered to reflect a high-level involvement with the child’s school duties but also with his general behavior. More specifically, the sample divided equally into 5 different age groups: 53 children (22.8%) aged 3 years (from 36 to 47 months), 49 children (21.1%) aged 4 years (from 48 to 59 months), 51 children (22%) aged 5 years (60 to 71 months), 57 children (24.6%) aged 6 years (72 to 83 months), and finally, 21 children (9.1%) aged 7 years (84 to 95 months). Respectively, these children attended the kindergarten, as well as the first two grades of the Primary School. The sample came from different state and private schools as well. Regarding the place of permanent residence, 158 children (68.1%) lived in a city, 33 children (14.7%) lived in a town, and 40 children (17.2%) lived in a village. All participants came from different educational and socioeconomic backgrounds from different regions of Greece.

At this point, it is essential to mention that, in order to test the psychometric properties of the assessment tools of the present study and taking into account the ages of the children to whom the tools were addressed, we administered both questionnaires, used in the present study, to parents (*N* = 108) of children aged 5 and 6 years of the sample. Parents (*N* = 102) of children aged 3 and 4 years were given and completed only the “Child Behavior Questionnaire”, whereas parents (*N* = 21) of children aged 7 years completed only the “Developmental Coordination Disorder Questionnaire”.

### 3.1. Instruments

#### 3.1.1. Children’s Behavior Questionnaire—Very Short Form (CBQ—VSF)

For The Child Behavior Questionnaire—Very Short Form (CBQ—VSF) [35], the scale assesses the behaviors of children aged 3 to 7 years. This questionnaire was developed based on the original Child Behavior Questionnaire (CBQ) [35]. Parents were asked to rate 36 sentences using a seven-point Likert scale (1 = extremely untrue for your child to 7 = extremely true for your child). In case a statement did not correspond to their child’s daily life or routine, the parents could respond with the alternative “Not applicable”, which exists after the scale, for such cases. Although the initial questionnaire scores consist of 15 scales, it’s very short form provides scores on three broad scales corresponding to Rothbart’s three main temperament factors: (1) Surgency/Extraversion, (2) Negative Affectivity, and (3) Effortful Control. Specifically, the Surgency/Extraversion scale includes 12 sentences (1, 4, 7, 10, 13, 16, 19, 22, 25, 28, 31, 34), which refer to the level of motor activity and pleasure of the child (e.g., “He always seems to be in a hurry to get from one place to another.”). The Negative Affectivity scale also includes 12 sentences (2, 5, 8, 11, 14, 17, 20, 23, 26, 29, 32, 35), which measure the feeling of fear, anger, sadness, and so on (e.g., “He gets quite frustrated when he is prevented from doing something he wants.”). Finally, the Effortful Control scale includes the remaining 12 sentences (3, 6, 9, 12, 15, 18, 21, 24, 27, 30, 33, 36), which evaluate the inhibitory control, the perceptual sensitivity, and so on (e.g., “When drawing or coloring in a book, it shows strong concentration”). For the purpose of the present research, the Greek version of the Child Behavior Questionnaire—Very Short Form (CBQ—VSF) was provided, which was edited by Mrs. Argyri and which is published on the official website of the questionnaire manufacturer. The test of the structural validity of this questionnaire has been carried out by Tsiara (2016) [36], during her dissertation thesis, based on the adaptation of the questionnaire by Kallia (2013) [37]. According to this paper, the Greek version of the Child Behavior Questionnaire—Very Short Form (CBQ—VSF) is structured in the three factors mentioned by the original psychometric tool, but 13 sentences out of the 36 included are excluded. Thus, while initially, a large number of factors were found in which the 36 sentences were charged, in the process of subtracting the sentences that were meaninglessly charged, the researcher came up with this analysis with 23 sentences instead of 36. More specifically, subtracting the sentences 1, 7, 11, 15, 16, 17, 25, 26, 27, 30, 31, 33, and 35, and then performing, with the method of Maximum Likelihood (ML), Confirmatory Factor Analysis for these 23 proposals, defining this time, the existence of the specific three factors according to Putnam and Rothbart [35], resulted in these 3 factors in a satisfactory adaptation to the Greek data. As a result of the analysis, Tsiara (2016) [36] ended up with the following sentences for every factor: for Surgency/Extraversion (with the sentences: 4, 10, 13, 19, 22, 28, 34, and *α* = 0.74), for Negative Affectivity (with the sentences: 2, 5, 8, 14, 20, 23, 29, 32, and *α* = 0.77), and for Effortful Control (with the sentences: 3, 6, 9, 12, 18, 21, 24, 36, and *α* = 0.71) [34].

#### 3.1.2. Developmental Coordination Disorder Questionnaire (DCDQ)

The Developmental Coordination Disorder Questionnaire (DCDQ) [38] is an assessment instrument developed to identify fine motor difficulties in children aged 5 to 14.6 years and is administered only to parents. This questionnaire requires parents to compare their child’s coordination status with other children of the same age and to rate it on a five-point Likert scale (1 = no similarity, 2 = slight similarity, 3 = moderate similarity, 4 = sufficient similarity, and 5 = great similarity). At this point, it is essential to mention that, in order to avoid parents’ biases, about half of the data are expressed negatively and half positively. Specifically, overall scores range from 17 to 85, with cut-off scores for “indication of disorder”, “suspicion of disorder”, or “probability of non-existence of the disorder”. The scores of this questionnaire created three different scales, which are: (1) Control during Movement, (2.) Fine Motor/Handwriting, and (3) General Coordination. More specifically, the Control during Movement scale includes sentences 1 to 6, the Fine Motor/Handwriting scale includes sentences 7 to 10, whereas the General Coordination scale includes the other 5 sentences, i.e., from 11 to 15. With Cronbach’s alpha *α* = 0.88, the internal consistency of the questionnaire is high. This questionnaire also demonstrates the validity of the construct by differentiating between children with and without Developmental Coordination Disorder (*F* (2,203) = 29.43, *p* < 0.001) and, through factor analysis, demonstrates that the scale measures motor skills in different environments [39,40].

Furthermore, for the purpose of the present study, the 15 proposals/items of the Developmental Coordination Disorder Questionnaire (DCDQ) [38] were translated from English into Greek. Specifically, the translation of the questionnaire into the Greek language was done by two of the authors (Georgia Papantoniou and Sophia Koulouri). For the translation of the DCDQ in Greek, the International Test Commission (ITC) guidelines (www.intestcom.org, accessed on 4 March 2021) were followed. The back translation procedure was also followed for the elimination of any inconsistencies that would disrupt the accuracy of the results [41]. The few differences that emerged in the back translation, compared to the original version, were taken into account in constructing the final form of the Greek version of the questionnaire.

## 4. Procedure

All parents participated in the study voluntarily. Each parent received a file, which contained: (a) the information letter concerning the objectives of the research, as well as the contact details of the research supervisor, (b) the form for completion of the demographic data, (c) the Greek version of the CBQ—VSF questionnaire, and (d) the Developmental Coordination Disorder Questionnaire (DCDQ) translated into Greek. All participants were examined individually and returned the signed file. Each parent had the opportunity to choose the place, as well as the time, to complete the questionnaires, while at the same time, there was the possibility for clarifications, where this was necessary. No time limit was assigned for the completion of the questionnaires. Through the information letter, parents were encouraged to respond honestly, so as to ensure the reliability of the results. Finally, the inclusion criteria for a child’s participation were: (a) the child has completed the third year of age but has not exceeded the eighth year of age, (b) the child attends a kindergarten, or one of the first two grades of primary school, and finally (c) the child has not been diagnosed with any type of psychiatric, neurological, or learning disorder.

Parents whose children met the inclusion criteria received a package containing an informative letter about the study and its purpose, and a consent form. All the participants were informed orally and in writing for the purpose of the study and had the opportunity to ask questions. They were also informed that their data would be confidentially collected in an electronic database. The participants gave written informed consent at the time of their visit, agreeing that their participation was voluntary and that they could withdraw at any time, without giving a reason and without cost. Due to the specific type of the current research, demographic data such as age, gender, or occupation were selected. Since these are considered personal data, the European Union law that exists since 28 May 2018 was applied. According to the law, the use of sensitive personal data is allowed only due to research reasons. Therefore, the participants were informed accordingly, and they also agreed that their personal data could be deleted from the web-database after a written request. The study’s protocol was approved by the Ethics Committee of the University of Ioannina in Greece, and followed the principles outlined in the Helsinki Declaration.

## 5. Results

Demographic characteristics of the professional and educational profile of parents are presented in Table 1.

### 5.1. Evaluation of the Psychometric Properties of the Greek Version of Children’s Behavior Questionnaire—Very Short Form (CBQ—VSF)

In order to verify the factor structure of the Greek CBQ—VSF that was proposed by Tsiara (2016) [36] in our sample, it was decided in the present study to carry out a confirmatory factor analysis based on the processing analyzed above. Before starting the data analysis process, the score was reversed in sentences 13, 19, 20, 22, 26, 29, 31, and 34, which had a negative wording. The implementation of the confirmatory factor analysis (CFA) was conducted in the statistical program EQS 6.1. [42] and, at first, was performed on a covariance matrix of the 23 items, according the research findings resulted in the initial evaluation of Tsiara (2016) [36], using the Maximum Likelihood estimation procedure. Our research findings contradicted with the findings of Tsiara (2016) [36]. More specifically, in the present study, there was a problem with the Surgency/Extraversion factor, which was not found to be statistically significant. In addition, the fitness indicators for the model were not acceptable: χ^2^ (230, *N* = 183) = 598.73, *p* < 0.000, CFI = 0.63, SRMR = 0.12, RMSEA = 0.09 (confidence interval (CI) 90% 0.08–0.10) [43,44,45]. Since, in the form of the 23 proposals of the questionnaire, the confirmatory factor analysis did not verify the Surgency/Extraversion factor, it was decided to carry out an exploratory factor analysis in order to investigate the structure of the questionnaire based on the original form of the 36 proposals. Initially, a sample suitability test for factor analysis was performed using the Kaiser-Meyer-Olkin (KMO) and Bartlett’s Test of Sphericity indicators. Thus, it appeared that: KMO = 0.69 (>0.50) and χ^2^ = 1,811,882, *df* = 630, *p* = 0.000. Going through the Varimax rotation analysis process, a large number of factors emerged in which the proposals were charged, as expected [36].

This was followed by the process of excluding improperly charged proposals, and at the same time, checking the reliability of internal consistency, given the existence of the three factors, as presented in the original, foreign form. Thus, for the factor of Surgency/Extraversion, the gradual exclusion of proposals and control of the credibility of the remaining ones resulted in 6 proposals. These are: 4, 7, 13, 16, 25, and 28, with Cronbach’s reliability coefficient *α* = 0.64. For the Negative Affectivity factor, following the same tactic, 8 sentences remained: 2, 5, 11, 14, 17, 23, 29, and 32, with α = 0.69. Finally, the Effortful Control factor ended up including+g 8 sentences, which are: 3, 6, 9, 12, 15, 18, 21, and 36, with Cronbach’s *α* = 0.78. The internal consistency reliability for the three subscales was acceptable.

As a result, this process led to a re-analysis of key components, in which only the 22 proposals mentioned above were taken into account, out of 36. Initially, Oblimin-type rotation was used to test for correlations between the three factors. As it turned out, these correlations are considered not statistically significant, and so, subsequently, a Varimax-type rotation was applied, during which a double charge of proposition 16 was presented on the first and third factor.

The test of the factor structure of the 21 other items (after the removal of the sentence 16) continued with the application of CFA through the statistical program EQS 6.1 [42]. The analysis began with the evaluation of the metric model, against which the three factors showed no correlations. The model was not acceptable according to various indices of fit: χ^2^ (189, *N* = 165) = 452.71, *p* < 0.000, CFI = 0.64, SRMR = 0.12, RMSEA = 0.09 (CI90% 0.08–0.10) [43,44,45]. Nevertheless, all parameters were found to be statistically significant in this model. Then, the structural model was checked, according to which there were correlations between the three factors. The indices in this model improved compared to the previous model but were still not acceptable: χ^2^ (186, *N* = 165) = 420.29, *p* < 0.000, CFI = 0.68, SRMR = 0.09, RMSEA = 0.08 (CI90% 0.07–0.09) [43,44,45]. However, the correlations between the factors appeared to be statistically significant. Then, we proceeded with the inclusion of the modifications indicated by the Wald and Lagrange Multiplier tests as well as the residual analyses into the final model, which achieved a marginally acceptable fit on all indices: χ^2^ (168, *N* = 165) = 237.28, *p* < 0.000, CFI = 0.90, SRMR = 0.07, RMSEA = 0.05 (CI90% 0.03–0.06) [43,44,45].

On the structure of the three factors of the Child Behavior Questionnaire, the three-factor model is presented in Table 2. The internal consistency reliability for the overall model was acceptable, with Cronbach’s *α* = 0.76.

### 5.2. Evaluation of the Psychometric Properties of the Greek Version of Developmental Coordination Disorder Questionnaire (DCDQ)

In order to evaluate the structural validity of the Greek version of the Developmental Coordination Disorder Questionnaire, a confirmatory factor analysis was conducted for the data collected from the 15 items that constitute it, in order to verify its three-factor structure, namely: (1) Control during Movement, (2) Fine Motor/Handwriting, and (3) General Coordination. The implementation of the CFA was conducted in the statistical program EQS 6.1. [42] and was performed on a covariance matrix of the 15 items, using the Maximum Likelihood estimation procedure. CFA began with the examination of the metric model, against which the three factors showed no correlations. The metric model was not acceptable according to various indices of fit: χ^2^ (90, *N* = 123) = 211.36, *p* < 0.000, CFI = 0.73, SRMR = 0.20, RMSEA = 0.10 (CI90% 0.08–0.12) [40,41,42]. Nevertheless, all parameters were found to be statistically significant in this model. Then, the structural model was checked, according to which there were correlations between the three factors. The indicators in this model were improved compared to the previous model and were marginally acceptable: χ^2^ (87, *N* = 123) = 126.09, *p* < 0.000, CFI = 0.91, SRMR = 0.06, RMSEA = 0.06 (CI90% 0.03–0.08) [43,44,45]. Furthermore, the correlations between the factors were statistically significant. Thus, the existence of the three factors, according to the original version of the questionnaire, was confirmed. Cronbach’s alpha coefficients of the Greek version of the Developmental Coordination Disorder Questionnaire were marginally acceptable to satisfactory. Specifically, for the overall DCDQ Cronbach’s *α* = 0.83, for Control during Movement scale *α* = 0.78, for Fine Motor/Handwriting scale *α* = 0.77, and for General Coordination scale *α* = 0.58.

### 5.3. Relations between the Temperament Components and Indicators of Developmental Coordination Disorder

In order to investigate the primary aim of the present study, the Pearson correlation coefficients between the research variables was estimated. As shown in Table 3, the temperament trait Effortful Control seems to be positively related to all three indicators of Motor Developmental Coordination Disorder, and Negative Affectivity was not found to be related to any motor coordination component. Additionally, temperamental Surgency/Extraversion was also not found to be related to Control during Movement.

In the first step of statistical analyses, in order to evaluate more precisely the causal relationships, according to the above-mentioned variables, a path analysis was conducted. Considering that path analysis—a structural equation modeling (SEM) technique for analyzing structural models with observed variables—is adequate for examining relationships among multiple constructs measured using summated scales [45,46], we proceeded with this analysis. Specifically, to examine the relationships between temperament components and indicators of developmental coordination disorder, a path analysis with manifest variables was computed. Because of the relatively small sample size, analysis was not run at the item level (observed variables). Instead, the covariance matrix was based on total scores (latent variables) for Surgency/Extraversion, Negative Αffectivity, Effortful Control, Control during Movement, Fine Motor/Handwriting, and General Coordination. The indicators of developmental coordination disorder were defined as endogenous variables. The three temperament components were defined as exogenous variables.

**Figure 1:**
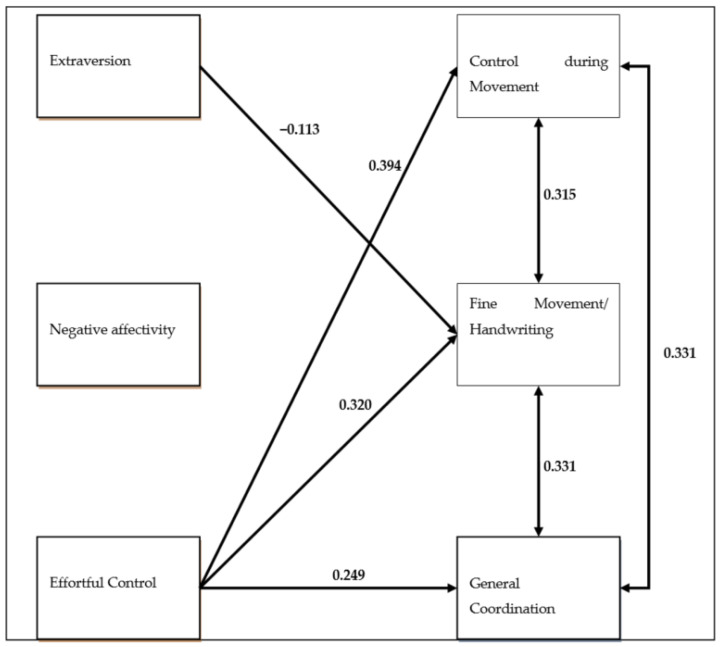
Path model displaying relationships between the components of temperament and the indicators of Developmental Coordination Disorder.

Path analysis was conducted in EQS 6.1 and was performed on a covariance matrix using the Maximum Likelihood estimation procedure [42]. As shown in Figure 1, Effortful Control was found to have a positive effect on all three indicators of Developmental Coordination Disorder. While in the path analysis, Surgency/Extraversion was not found to affect Control during Movement, and as expected, an unexpected low negative relationship emerged between Surgency/Extraversion and Fine Movement/Handwriting style. Finally, Negative Affectivity was not found to affect any index of the Developmental Coordination Disorder. The indices of this path model were excellent: χ^2^ (3, *N* = 88) = 0.522, *p* < 0.000, CFI = 1.00, SRMR = 0.01, RMSEA = 0.00 (CI90% 0.00–0.06). The model is presented in Figure 1.

### 5.4. Investigating Gender Differences in the Three Components of Temperament

In order to test the third aim of this study, one-way analysis of variance (ANOVA) was applied between the three temperament factors found in the analysis of the structure of the Child Behavior Questionnaire as well as in the girl and boy groups, as shaped for the ages of the sample (3–7 years). As ANOVA (*F* (1187) = 10.951, *p* < 0.001) revealed, only the Effortful Control seemed to differentiate between boys and girls, with the latter showing a higher value (M = 45.05 and SD = 8.08).

## 6. Discussion

This study addressed relations between Developmental Coordination Disorder and temperament early in life. Moreover, a significant purpose of the current research was the evaluation of the structural validity of the Greek versions of the Child Behavior Questionnaire (CBQ—VSF) and for the Developmental Coordination Disorder Questionnaire (DCDQ) in parents of children in early childhood (preschool and early school age).

### 6.1. Evaluation of the Psychometric Properties of the Greek Version of Children’s Behavior Questionnaire (CBQ)

The very short form of the Children’s Behavior Questionnaire (CBQ—VSF) [35] was administered to parents in order to measure the temperamental characteristics of their children of these ages. A series of exploratory and confirmatory factor analyses was conducted in order to verify the three broad factors. Regarding the fact that this process was carried out in the past by Tsiara (2016) [36], in the present study, an attempt was made to confirm the specific resulting structure, in which the 3 factors emerge after the exclusion of 13 sentences from the 36 included in the questionnaire.

The included sentences of the initial questionnaire for the analysis were: 2, 3, 4, 5, 6, 8, 9, 10, 12, 13, 14, 18, 19, 20, 21, 22, 23, 24, 28, 29, 32, 34, and 36. In the present study, however, this structure was not found to agree with previous findings [36]. In fact, in the context of the test of the structure, it was necessary to exclude more statements of the questionnaire. As a result, the proposals that were removed were finally 15 out of 36, which differed from the proposals mentioned above. Thus, the ones that finally remained based on the confirmatory analysis were the proposals: 2, 3, 4, 5, 6, 7, 9, 11, 12, 13, 14, 15, 17, 18, 21, 23, 25 28, 29, 32, and 36. In fact, only 16 of the 21 proposals in this work model were the same ones included in Tsiara’s (2016) research work. This may be due to the fact that in the present study, the same form of the Greek version of the questionnaire was not used as that of Tsiara (2016) [36]. The work of Tsiara [36] was based on the adaptation of the questionnaire by Kallia (2013) [37], while in the present work, the translation of the version of the very short form of the Children’s Behavior Questionnaire was provided, which was edited by Mrs. Argyri. We do not consider the two translations of the questionnaire in Greek to be particularly distant from each other, however the lack of complete agreement of the findings may be attributed to any detailed difference in translation, as the children age groups and sample size are in line between the two surveys.

It is logical to consider that the process of excluding items of the Children’s Behavior Questionnaire, which was applied both in the present and in Tsiara’s work (2016) [36], was obviously necessary, as it is consistent with previous findings [32], in which if the indicators are modified, a satisfactory adjustment of the CBQ structure control model will occur. Furthermore, Gouze, Lavigne, Hopkins, Bryant, and Lebailly (2012) [47] also confirmed this statement, through the application of factor analysis in order to control the original Rothbart model (2001) regarding the broad factors of “Negative Affectivity” and “Effortful Control”. They also found that, without modifications to its indicators, the original model eventually had a poor fit.

### 6.2. Evaluation of the Psychometric Properties of the Greek Version of Developmental Coordination Disorder Questionnaire (DCDQ)

The assessment instrument chosen to measure and evaluate the indicators of Neurodevelopmental Coordination Disorder is the Developmental Coordination Disorder Questionnaire [35]. The confirmatory factor analysis applied to this tool also revealed that, as in the original version, the Greek version of the DCDQ, which is addressed to parents, assesses the subtle motor difficulties in children, as they are structured in three factors: Movement control (Control During Movement), Fine Motor/Handwriting, and General Coordination.

Based on the above results, the DCDQ questionnaire was found to fully retain the factorial structure proposed by its manufacturers, when administered to the sample of the present study. This is an encouraging indication of its future normative data and use in the Greek population as an assessment tool for evaluating and identifying symptoms of Developmental Coordination Disorder in young children.

### 6.3. Relations between the Temperament Components and Indicators of Developmental Coordination Disorder

Concerning the evaluation of the relationships between temperament components and Developmental Coordination Disorder, through path analysis, the effortful control was found, as expected, in an attempt to have a positive effect on the lack of all three indicators regarding the Developmental Coordination Disorder. This self-regulating ability as a temperamental component mirrors both inhibitory and stimulatory abilities, that is, the child’s ability to actively suppress or delay the approach, as well as the ability to initiate an activity. Furthermore, it shapes the child’s attention and behavior, such as the ability to manage rhythm and control impulses [14,47]. This is also consistent with the finding of Nakagawa et al. (2016) [26] and indicates that high temperamental Effortful Control (efficient executive functions) may compensate for atypicalities in other brain systems in early childhood.

In line with the above, motor coordination is associated with behavioral and neurological markers of neurodevelopmental disorders and appears to be influenced by Effortful Control [17]. This finding is closely aligned with the research point of view according to which high effortful control or high performance of executive attention can offset the peculiarities of other brain systems at the beginning of life. Temperamental Effortful Control could therefore be defined as the efficiency of the neural network involved in executive attention [48].

On the other hand, the Surgency/Extraversion factor was not found to affect the control during movement. Extraversion highlights the willingness to approach potentially enjoyable activities. It is possible that the research finding regarding lack of relationship between extraversion and control in motion is due to the fact that extraversion manifests itself in the stage of approach, opposite to control in motion, which manifests itself in the act of movement and control [25]. However, based on path analysis, an unexpected low and negative relationship emerged between the Surgency/Extraversion and the Fine Motor/Handwriting, as extraversion was found to adversely affect fine mobility. This research finding may be due to the fact that a child’s need to be able to respond to fine motor skills is inhibited by the need to express emotions in order to unwind or by the willingness to approach potentially enjoyable activities [47,49,50,51]. Additionally, as expected, in the analysis of pathways between the components of temperament and Developmental Coordination Disorder, negative mood was not found to affect any of the DCDQ factors [26].

### 6.4. Gender Differences

Moreover, analysis of variance revealed that only effortful control seemed to differentiate between boys and girls, with the latter showing a higher value. This finding was expected, regarding the levels of the Negative Affectivity as a temperamental trait did not appear to differ between the two groups. The literature review reveals sporadic evidence for gender differences in temperament from infancy [32]. Furthermore, in line with the above, researchers noted that up to 18 months, boys and girls are rated similarly in emotional upsets and frustration reactions. After 18 months of age, boys show more negative emotional outbursts [52]. On the contrary, in late adolescence, girls show more emotional reactivity than do boys [53]. At this point, it is essential to mention that as temperament develops, gender differences in temperament may be attenuated by significant moderating factors such as the age of the child, the source of the temperament assessment (e.g., mother or teacher report), cultural and socioeconomic contexts, and whether the children are drawn from a special population (e.g., children at risk for behavioral disorders). The literature review reveals several studies indicating that young girls are more likely to comply with prohibitions and requests, rather than boys. This result could have been influenced by differential socialization of self-control in boys and girls. Furthermore, it is essential to mention that individual differences in children’s Effortful Control may also contribute to sex difference findings [25]. More research is needed in order to identify gender differences regarding the developmental nature of temperament.

## 7. Conclusions

Under the aegis of our research findings, this work could be seen as a preliminary attempt to evaluate the relationships between temperamental traits and the indicators of Developmental Coordination Disorder. The current findings show Effortful Control as the main and significant factor influencing all DCDQ indicators, while the Surgency/Extraversion had an unpredictable low and negative effect on Fine Motor/Handwriting. This research has important clinical and practical implications. Specifically, the evaluation of the complex relationship between temperament of characteristics and the indicators of Developmental Coordination Disorder can be transformed into an innovative screening instrument by providing early identification and timely targeted intervention. Additionally, the early identification may lead to essential support for these children.

Although more research is also needed to further validate and refine the Greek versions of the Children’s Behavior Questionnaire (CBQ) and the Developmental Coordination Disorder Questionnaire, and to replicate our current findings, the results of this study, with the size of the sample used, show that the Greek versions of the aforementioned questionnaires are useful and valid instruments for measuring temperament components and indicators of Developmental Coordination Disorder, in the Greek cultural context, and their existence can extend evolving cross-cultural endeavors on research. The continued use of these questionnaires within the potential links with temperament and DCD can enhance questionnaires’ adaptation for other cultures in order to understand and illuminate the complex relationship of motor difficulties and temperament in young children. Finally, the early identification can lead to encourage the participation in typical activities, regarding education, during childhood. As a result, early intervention can decrease the risk of low self-esteem and social participation and interaction. Moreover, identification at an earlier age may improve the psychosocial and educational outcomes of children with DCD.

## Figures and Tables

**Table 1:** Educational and professional profile of parents.

Years of Education	*N*	Frequency (%)	Professional Status	*N*	Frequency (%)
Basic Education	10	4.3%	Unemployed	24	10.4%
Secondary Education	69	29.9%	Professional Scientists	15	6.5%
University Degree	117	50.6%	Scientists Employees	69	29.9%
Masters’ Degree	25	10.8%	State/Private Employees	78	33.8%
PhD	10	4.3%	Businessmen	19	8.2%
			Farmers	2	0.9%
			Engaged in Household	24	10.4%

**Table 2:** The structure of the revised Children’s Behavior Questionnaire—Very Short Form (CBQ—VSF) (standardized solution).

Statements	Extraversion	Negative Affectivity	Effortful Control	Error	*R* ^2^
Likes going down high slides or other adventurous activities (Statement 4)	0.653			0.757	0.427
Often rushes into new situations (Statement 7)	0.553			0.833	0.305
Prefers quiet activities to active games (Statement 13)	0.360			0.933	0.130
Is full of energy, even in the evening (Statement 25)	0.451			0.893	0.203
Likes rough and rowdy games (Statement 28)	0.403			0.915	0.162
Gets quite frustrated when prevented from doing something s/he wants to do (Statement 2)		0.539		0.842	0.291
Is quite upset by a little cut or bruise (Statement 5)		0.443		0.897	0.196
Is afraid of burglars or the “boogie man” (Statement 11)		0.325		0.946	0.106
When angry about something, s/he tends to stay upset for ten minutes or longer (Statement 14)		0.604		0.797	0.365
Seems to feel depressed when unable to accomplish some task (Statement 17)		0.580		0.815	0.336
Is very difficult to soothe when s/he has become upset (Statement 23)		0.667		0.745	0.445
Is not very upset at minor cuts or bruises (Statement 29)		0.202		0.979	0.041
Is slow and unhurried in deciding what to do next (Statement 32)		0.453		0.892	0.205
When drawing or coloring in a book, shows strong concentration (Statement 3)			0.664	0.747	0.441
Prepares for trips and outings by planning things s/he will need (Statement 6)			0.385	0.923	0.148
Likes being sung to (Statement 9)			0.394	0.919	0.155
Notices it when parents are wearing new clothing (Statement 12)			0.579	0.815	0.335
When building or putting something together, becomes very involved in what s/he is doing, and works for long periods (Statement 15)			0.459	0.888	0.211
Is good at following instructions (Statement 18)			0.566	0.824	0.321
Likes the sound of words, such as nursery rhymes (Statement 21)			0.572	0.820	0.327
Comments when a parent has changed his/her appearance (Statement 36)			0.439	0.898	0.193
**Correlations between factors (latent variables)**	**Correlations between the items’ (observed variables’) measurement errors**				
F1 − F2 = 0.276	E3 − E2 = 0.210				
F3 − F1 = 0.262	E12 − E2 = 0.363				
F3 − F2 = 0.442	E36 − E2 = 0.240				
	E17 − E3 = 0.215				
	E28 − E3 = −0.340				
	E6 − E4 = 0.365				
	E29 − E5 = 0.408				
	E7 − E6 = 0.371				
	E13 − E6 = −0.217				
	E21 − E9 = 0.322				
	E28 − E9 = −0.198				
	E17 − E12 = 0.328				
	E36 − E12 = 0.462				
	E17 − E15 = 0.287				
	E13 − E15 = −0.188				
	E36 − E17 = 0.215				
	E23 − E18 = −0.195				
	E13 − E18 = −0.185				

Note 1: E = Items’ Measurement Errors Correlations. *R*^2^ = Multiple correlation coefficient raised to the square. F1 = Effortful Control. F2 = Negative Affectivity. F3 = Extraversion. Note 2: All model parameters are statistically significant at the *p* < 0.05 level.

**Table 3:** Correlations between temperament components and indicators of Developmental Coordination Disorder.

	Extraversion	Negative Affectivity	Effortful Control	Control during Movement	Fine Motor/Handwriting	General Coordination
Extraversion						
Negative Affectivity	0.237 **					
Effortful Control		0.249 **				
Control during Movement			0.408 **			
Fine Movement			0.316 **	0.505 **		
General Coordination			0.266 *	0.486 **	0.422 **	

* Correlation is significant at the 0.05 level (2-tailed). ** Correlation is significant at the 0.01 level (2-tailed).

## Data Availability

The data presented in this study are available on request from the corresponding author. The data are not publicly available due to privacy.
